# Trends in Alcohol-Related Deaths by Sex in the US, 1999-2020

**DOI:** 10.1001/jamanetworkopen.2023.26346

**Published:** 2023-07-28

**Authors:** Ibraheem M. Karaye, Nasim Maleki, Nawaal Hassan, Ismaeel Yunusa

**Affiliations:** 1 Department of Population Health, Hofstra University, Hempstead, New York; 2 Department of Psychiatry, Harvard Medical School, Massachusetts General Hospital, Charlestown; 3 Clinical Pharmacy and Outcomes Sciences, University of South Carolina, Columbia

## Abstract

**Importance:**

Alcohol consumption rates have been increasing among women in the US, which may affect mortality rates and sex gaps. Therefore, conducting a comprehensive assessment of sex differences in alcohol-related deaths is essential to inform targeted interventions and policies aimed at reducing the burden of alcohol-related harm among the population.

**Objective:**

To examine sex differences in the burden and trends of alcohol-related mortality in the US from 1999 to 2020.

**Design, Setting, and Participants:**

This cross-sectional time series study used Centers for Disease Control and Prevention Wide-Ranging Online Data for Epidemiologic Research data on alcohol-related deaths from 1999 to 2020. Alcohol-related deaths were identified from the underlying cause of death files using *International Statistical Classification of Diseases and Related Health Problems, Tenth Revision*, codes, including alcohol-related poisoning, liver disease, gastritis, cardiomyopathy, myopathy, polyneuropathy, and pseudo-Cushing syndrome, among others.

**Main Outcomes and Measures:**

Age-adjusted mortality rates (AAMRs) were analyzed by sex and substratified by race and ethnicity, age, and census region. Rate ratios and 95% CIs calculated by Taylor series were used to assess sex differences in mortality burden. Joinpoint regression was used to assess temporal trends.

**Results:**

A total of 605 948 alcohol-attributed deaths were identified in the US from 1999 through 2020 (AAMR, 8.3 per 100 000 persons; 95% CI, 8.3-8.3 per 100 000 persons). The mortality burden was higher among male individuals than female individuals, with male individuals being 2.88 (95% CI, 2.86-2.89) times more likely to die compared with female individuals. However, temporal trends showed an increase in alcohol-related deaths for both male and female individuals in recent years, with higher rates of increase among female individuals relative to male individuals. The AAMR increased by 12.5% (95% CI, 6.4%-19.1%) per year among male individuals from 2018 to 2020 but increased by 14.7% (95% CI, 9.1%-20.5%) per year among female individuals during the same period. Trend differences were observed across subtypes of age, race and ethnicity, cause, and region.

**Conclusions and Relevance:**

This study of alcohol-related mortality in the US suggests there has been a significantly higher rate of increase in deaths among female individuals in recent years. These findings underscore the need for further research to understand the specific factors associated with this trend. The development of targeted interventions and evidence-based treatments for alcohol use among female individuals becomes imperative in effectively addressing the increasing rates of alcohol-related deaths.

## Key Points

**Question:**

Are there sex-based differences in the contemporary burden and trends of alcohol-related mortality in the US?

**Findings:**

In this cross-sectional study of 605 948 alcohol-attributed deaths, male individuals had a significantly higher burden of alcohol-involved mortality than did female individuals, with a male to female ratio of 2.88. Temporal trends revealed an increase in alcohol-related deaths among both sexes, with a significantly higher rate of increase observed for female individuals than for male individuals.

**Meaning:**

Although alcohol-related deaths have historically been more prevalent among men than women, recent temporal trends suggest a narrowing of this gap, with increasing rates of alcohol-related deaths among female individuals compared with male individuals.

## Teaser

This cross-sectional study uses data from Centers for Disease Control and Prevention Wide-Ranging Online Data for Epidemiologic Research to examine sex differences in the burden and trends of alcohol-related mortality in the US from 1999 to 2020.

## Introduction

Recently, the World Health Organization declared that even small amounts of alcohol consumption are detrimental to human health.^1^ In the US, alcohol ranks as the fourth leading cause of preventable death, trailing tobacco, poor diet and physical inactivity, and illegal drugs, resulting in more than 140 000 deaths annually.^2,3^ Alcohol is implicated in 18.5% of emergency department visits and 20% of prescription opioid deaths.^2,4^

Although alcohol consumption is associated with adverse health outcomes, the distribution of harm varies across the US population.^5,6^ Sex differences in alcohol-related complications have been observed, with a historically greater burden among men than women.^7^ However, recent studies indicate a narrowing sex gap, attributed in part to increased alcohol use, high-risk drinking, and alcohol use disorder (AUD) among women.^8,9,10,11,12^ This reversal raises public health concerns because heightened alcohol consumption among women may be associated with elevated complications due to metabolic and physiological differences.

Women tend to have a higher percentage of body fat and a lower percentage of body water compared with men, resulting in higher alcohol blood concentrations and potentially increasing vulnerability to complications.^13,14^ Hormonal fluctuations throughout the menstrual cycle can influence alcohol processing, with certain phases heightening sensitivity to alcohol’s effects.^15,16^ Women also have lower levels of alcohol-metabolizing enzymes, such as alcohol dehydrogenase, leading to slower alcohol metabolism, prolonged exposure to harmful byproducts (such as acetaldehyde), and potentially more severe physiological and organ damage over time.^17,18^ Consequently, women with AUD face an elevated risk of developing liver diseases, circulatory disorders, breast cancer, fertility problems, and early menopause.^10,14^

Although recent studies note a narrowing sex gap in alcohol-related harm, it remains unclear whether this convergence extends to alcohol-related death rates. The existing literature has limitations, including using secondary designs^10^ and outdated data^5,11^ or focusing on nonmortality variables, such as alcohol consumption or alcohol-associated liver disease.^19^ In addition, some studies have primarily examined short-term changes associated with the COVID-19 pandemic.^20,21^ For instance, Angus et al^20^ explored “deaths of despair,” including alcohol-related mortality, with a specific focus on the association between the COVID-19 pandemic and mortality. Similarly, White et al^21^ examined recent data emphasizing the association between COVID-19 and alcohol-related deaths. Given the public health significance of alcohol and the reported changes in female alcohol consumption, there is a need to conduct a comprehensive assessment of sex differences in alcohol-associated deaths using contemporary data. This study aims to use recent national mortality data from the National Center for Health Statistics, assessing sex differences in alcohol-related mortality within the US from 1999 to 2020.

## Methods

### Data Sources

This study followed the Strengthening the Reporting of Observational Studies in Epidemiology (STROBE) reporting guideline for cross-sectional studies.^22^ We obtained national mortality data from the Centers for Disease Control and Prevention Wide-ranging Online Data for Epidemiologic Research (CDC WONDER).^23^ The *International Statistical Classification of Diseases and Related Health Problems, Tenth Revision* (*ICD-10*) codes E24.4, F10, G31.2, G62.1, G72.1, I42.6, K29.2, K70, K85.2, K86.0, R78.0, X45, X65, and Y15 were used to identify alcohol-related deaths recorded in the US between 1999 and 2020. Alcohol-induced causes specifically exclude deaths resulting from unintentional injuries, homicides, and other causes of death that are only indirectly or partially associated with alcohol use.^24^ A summary list of the *ICD-10* codes, along with their descriptions and distributions, is provided in the eTable in Supplement 1. This study was deemed exempt from review and the requirement for informed consent by the Hofstra University institutional review board because the data obtained from the CDC WONDER are deidentified and publicly available.

We defined the study period as being from 1999 to 2020 based on data availability, with 2020 being the most recent year for which data are accessible. Crude or age-adjusted mortality rates (AAMRs) were abstracted by age (15-24, 25-44, 45-64, or ≥65 years), sex (male or female), race and ethnicity (American Indian or Alaska Native, Asian or Pacific Islander, Hispanic, non-Hispanic Black, or non-Hispanic White), cause of death (alcohol poisoning, alcoholic liver disease, mental and behavioral disorders due to use of alcohol, or other), and census region (Northeast, Midwest, South, or West).

Race and ethnicity data of the deceased individuals were collected from their death certificates, adhering to the guidelines set by the Office of Management and Budget.^23^ The information documented on the death certificate relied primarily on the input of the funeral director, a report by an informant or, in the absence of an informant, physical observation. Sex information is recorded as “male” or “female” in CDC WONDER and reflects mortality data obtained from death certificates.^25^ These certificates are collected by state registries and subsequently shared with the National Vital Statistics System for US residents.^25^

Including race and ethnicity in this study was essential for multiple reasons. First, alcohol-related mortality burden exhibits significant variation among different racial and ethnic groups, reflecting disparities in alcohol consumption patterns, health care access, socioeconomic factors, and cultural influences.^6^ By using race and ethnicity as stratification variables, we investigated whether distinct patterns of alcohol-related mortality existed across these groups.

Second, examining the association of race and ethnicity with alcohol-related mortality allows us to gain a better understanding of the social determinants of health and identify potential health disparities within the population. This information plays a crucial role in formulating targeted interventions and policies to address these disparities and foster health equity.^6^

Finally, incorporating race and ethnicity as variables in our analysis facilitated exploration of potential interactions or modifying effects between these factors and sex. These interactions may offer insights into the complex relationships and underlying mechanisms contributing to alcohol-related mortality disparities.

### Statistical Analysis

We calculated the sex-based mortality rate ratios by dividing the mortality rate among male individuals by the mortality rate among female individuals. The 95% CIs for these estimates were derived using the Taylor series method. This method was selected (eg, instead of Poisson regression) due to its simplicity and computational efficiency, facilitating direct estimation of the sex-based mortality rate ratios. We assessed temporal trends in AAMRs using joinpoint regression, a statistical technique that initially assumes a linear trend in AAMR throughout the study period. We then added a joinpoint to signify an inflection point (ie, change in trend) and used the permutation test to assess the significance of this joinpoint relative to the initial null model.^26^ If the joinpoint was significant, we retained it; otherwise, we excluded it from the analysis. We repeated these steps, using the Bonferroni correction for multiple testing, until an optimum number of joinpoints was obtained from 4499 Monte Carlo permutations—the default Monte Carlo sample of permuted data sets.^27,28^ We derived 95% CIs using the parametric method. All statistical analysis was conducted using the Joinpoint Regression Program, version 4.9.1.0 (Division of Cancer Control & Population Sciences, National Cancer Institute), OpenEpi, version 3.01 (Open Source Epidemiologic Statistics for Public Health), and Stata, version 17.0 (StataCorp LP).

To account for the potential association of the COVID-19 pandemic with alcohol-related mortality rates, we conducted a sensitivity analysis by excluding data from the year 2020. This approach allowed us to examine the trends in alcohol-related deaths before the onset of the pandemic and evaluate the robustness of our findings.

We modeled the log-transformed AAMR as a function of the year of death. To account for heteroscedasticity or correlated errors, we confirmed constant variance using the Breusch-Pagan test and fitted an uncorrelated errors model. We imputed the interval type as “annual” to permit yearly trend estimations. We used default options for the method (grid search), the number of joinpoints (0-4), the model selection method (permutation test), the overall significance level (*P* < .05), the number of permutations (4499), the average annual percentage change segment ranges (entire range), the annual percentage change (APC), the average annual percentage change, and the tau confidence intervals (parametric method).

## Results

Between 1999 and 2020, a total of 605 948 individuals died in the US due to alcohol-related causes, resulting in an AAMR of 8.3 per 100 000 persons (95% CI, 8.3-8.3 per 100 000 persons). Men had a significantly higher mortality rate compared with women, with men being 2.88 (95% CI, 2.86-2.89) times more likely to die from alcohol-related causes. This sex disparity in alcohol-related mortality burden persisted across various subcategories, including age, race and ethnicity, census region, and cause of death (Table 1).

**Table 1.** Sex Differences in Alcohol Mortality Rates in the US by Race and Ethnicity, Age, Region, and Cause, 1999-2020

Variable	AAMR (95% CI), %, per 100 000 persons	AAMR (95% CI), %, per 100 000 persons		Mortality rate ratio (95% CI)^a^
		Male	Female	
Overall	8.3 (8.3-8.3)	12.7 (12.7-12.7)	4.3 (4.3-4.3)	2.88 (2.86-2.89)^b^
Race and ethnicity^c^				
American Indian or Alaska Native	38.7 (38.1-39.2)	50.9 (50.0-51.8)	27.6 (27.0-28.2)	1.76 (1.71-1.81)^b^
Asian or Pacific Islander	2.0 (1.9-2.0)	3.5 (3.4-3.6)	0.7 (0.7-0.7)	4.59 (4.33-4.88)^b^
Black	7.1 (7.1-7.2)	11.6 (11.5-11.8)	3.6 (3.5-3.6)	2.91 (2.85-2.96)^b^
Hispanic	9.7 (9.6-9.8)	16.9 (16.7-17.0)	3.2 (3.1-3.3)	4.70 (4.62-4.79)^b^
White	8.4 (8.4-8.4)	12.4 (12.4-12.5)	4.6 (4.6-4.6)	2.71 (2.69-2.72)^b^
Age, y				
15-24	0.3 (0.3-0.4)	0.5 (0.5-0.5)	0.2 (0.2-0.2)	2.98 (2.75-3.23)^b^
25-44	5.8 (5.8-5.9)	8.2 (8.1-8.2)	3.5 (3.5-3.6)	2.30 (2.27-2.33)^b^
45-64	21.5 (21.5-21.6)	32.5 (32.3-32.6)	11.1 (11.0-11.2)	2.96 (2.93-2.98)^b^
≥65	13.7 (13.7-13.8)	23.6 (23.4-23.7)	6.0 (5.9-6.1)	4.15 (4.10-4.21)^b^
Census region				
Northeast	6.2 (6.1-6.2)	9.6 (9.6-9.7)	3.1 (3.0-3.1)	3.05 (3.00-3.10)^b^
Midwest	7.5 (7.5-7.5)	11.4 (11.3-11.5)	3.9 (3.9-4.0)	2.87 (2.84-2.91)^b^
South	7.4 (7.3-7.4)	11.6 (11.5-11.7)	3.6 (3.6-3.6)	3.14 (3.11-3.17)^b^
West	12.4 (12.3-12.4)	18.3 (18.2-18.4)	6.9 (6.8-6.9)	2.56 (2.53-2.58)^b^
Cause				
Alcohol poisoning (overdose)	0.5 (0.5-0.5)	0.8 (0.8-0.8)	0.2 (0.2-0.3)	3.23 (3.15-3.31)^b^
Alcoholic liver disease	5.1 (5.1-5.1)	7.5 (7.5-7.6)	2.9 (2.9-2.9)	2.55 (2.53-2.57)^b^
Mental and behavioral disorders due to use of alcohol, acute intoxication	2.4 (2.4-2.4)	3.8 (3.8-3.9)	1.0 (1.0-1.1)	3.50 (3.46-3.54)^b^
All other causes	0.3 (0.3-0.3)	0.6 (0.5-0.6)	0.1 (0.1-0.1)	4.63 (4.48-4.78)^b^

Abbreviation: AAMR, age-adjusted mortality rate.

^a^ Calculated by dividing the AAMR among male individuals by the AAMR among female individuals. The 95% CIs were derived using the Taylor series.

^b^ *P* < .05; 95% CI does not include 1.

^c^ Hispanic individuals could be of any race; all other categories are non-Hispanic.

### Sex Differences in Alcohol-Related Mortality Trends

Overall alcohol-related mortality trends in the US were stable from 1999 to 2007 (APC, 0.0; 95% CI, −0.6 to 0.6) but increased by 3.0% per year (95% CI, 2.6%-3.5%) from 2007 to 2018 and, more recently, by 14.1% per year (95% CI, 8.2%-20.3%) from 2018 to 2020.

When examining sex-specific trends, male individuals had a stable trend from 1999 to 2009 (APC, 0.0; 95% CI, −0.5 to 0.4), with increasing trends at an annual rate of 3.0% (95% CI, 2.4%-3.6%) from 2009 to 2018 and 12.5% (95% CI, 6.4%-19.1%) from 2018 to 2020. Female individuals had a slightly different trend, with a 1.0% (95% CI, 0.4%-1.5%) per year increase from 1999 to 2007, followed by a larger increase of 4.3% (95% CI, 3.9%-4.8%) per year from 2007 to 2018, and an even larger increase of 14.7% (95% CI, 9.1%-20.5%) per year from 2018 to 2020 (Figure).

**Figure.**
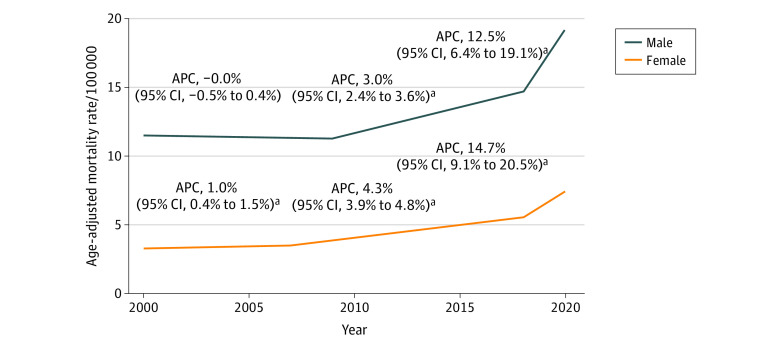
Temporal Trends in Alcohol-Related Mortality Rates by Sex, US, 1999-2020 APC indicates annual percentage change. ^a^*P* < .05; 95% CI does not include zero.

When stratified by age and sex, alcohol-related mortality trends were found to be increasing among both male and female individuals across all age groups. However, among individuals younger than 60 years, the rate of increase in the most recent trend was higher among male individuals than female individuals. Among adults aged 65 years or older, female individuals showed a higher annual rate of change compared with male individuals (6.7% [95% CI, 5.4%-8.0%] per year vs 5.2% [95% CI, 4.3%-6.1%] per year) from 2012 to 2020 (Table 2). The temporal trends for female individuals aged 15 to 24 years were not evaluated due to limited and unreliable data counts.

**Table 2.** APCs and AAPCs in Alcohol-Related Mortality Rates by Sex, US, 1999-2020

Trend segment	Segment end points, y		APC (95% CI), %^a^	AAPC (95% CI), %^b^
	Lower	Upper		
**Overall**				
1	1999	2007	0.0 (−0.6 to 0.6)	2.9 (2.3 to 3.4)^c^
2	2007	2018	3.0 (2.6 to 3.5)^c^	
3	2018	2020	14.1 (8.2 to 20.3)^c^	
**Sex**				
Male				
1	1999	2009	−0.0 (−0.5 to 0.4)	2.4 (1.8 to 3.0)^c^
2	2009	2018	3.0 (2.4 to 3.6)^c^	
3	2018	2020	12.5 (6.4 to 19.1)^c^	
Female				
1	1999	2007	1.0 (0.4 to 1.5)^c^	4.0 (3.5 to 4.5)^c^
2	2007	2018	4.3 (3.9 to 4.8)^c^	
3	2018	2020	14.7 (9.1 to 20.5)^c^	
**Age and sex**				
15-24 y				
Male				
1	1999	2008	7.2 (4.0 to 10.4)^c^	2.4 (−0.9 to 5.9)
2	2008	2018	−5.4 (−8.1 to −2.5)^c^	
3	2018	2020	23.9 (−10.4 to 71.4)	
25-44 y				
Male				
1	1999	2009	−1.4 (−2.2 to −0.7)^c^	3.1 (2.0 to 4.1)^c^
2	2009	2018	3.5 (2.4 to 4.6)^c^	
3	2018	2020	26.8 (14.9 to 39.9)^c^	
Female				
1	1999	2010	−0.0 (−0.8 to 0.8)	4.4 (3.1 to 5.6)^c^
2	2010	2018	6.5 (4.8 to 8.1)^c^	
3	2018	2020	22.1 (8.8 to 36.9)^c^	
45-64 y				
Male				
1	1999	2006	0.3 (−0.2 to 0.8)	2.3 (1.9 to 2.7)^c^
2	2006	2018	2.3 (2.0 to 2.5)^c^	
3	2018	2020	10.0 (6.0 to 14.2)^c^	
Female				
1	1999	2007	2.7 (1.5 to 3.9)^c^	4.0 (3.5 to 4.6)^c^
2	2007	2020	4.8 (4.3 to 5.4)^c^	
≥65 y				
Male				
1	1999	2003	−2.9 (−5.3 to −0.5)^c^	1.6 (1.0 to 2.2)^c^
2	2003	2012	0.5 (−0.3 to 1.4)	
3	2012	2020	5.2 (4.3 to 6.1)^c^	
Female				
1	1999	2012	−0.4 (−1.0 to 0.2)	2.2 (1.7 to 2.8)^c^
2	2012	2020	6.7 (5.4 to 8.0)^c^	
**Race and ethnicity and sex^d^**				
American Indian or Alaska Native				
Male				
1	1999	2006	−0.0 (−2.8 to 2.9)	2.8 (1.7 to 3.9)^c^
2	2006	2020	4.2 (3.2 to 5.3)^c^	
Female				
1	1999	2018	3.9 (3.5 to 4.4)^c^	5.6 (4.2 to 7.0)^c^
2	2018	2020	22.8 (5.9 to 42.4)^c^	
Asian or Pacific Islander				
Male				
1	1999	2016	1.2 (0.3 to 2.1)^c^	2.9 (1.3 to 4.5)^c^
2	2016	2020	10.4 (2.0 to 19.5)^c^	
Female				
1	1999	2003	−10.6 (−22.5 to 3.2)	2.2 (−0.6 to 5.1)
2	2003	2020	5.5 (3.8 to 7.2)^c^	
Black				
Male				
1	1999	2008	−5.7 (−6.7 to −4.7)^c^	−0.6 (−1.8 to 0.5)
2	2008	2018	1.0 (−0.0 to 2.1)	
3	2018	2020	15.8 (3.3 to 29.7)^c^	
Female				
1	1999	2007	−5.8 (−7.4 to −4.2)^c^	0.7 (−0.9 to 2.3)
2	2018	2018	2.8 (1.6 to 4.1)^c^	
3	2020	2020	17.0 (0.3 to 36.4)^c^	
Hispanic				
Male				
1	1999	2012	−1.5 (−2.2 to −0.8)^c^	0.2 (−0.5 to 0.9)
2	2012	2020	3.1 (1.6 to 4.7)^c^	
Female				
1	1999	2005	−1.7 (−5.2 to 1.8)	1.50 (0.4 to 2.7)^c^
2	2005	2020	2.9 (2.0 to 3.8)^c^	
White				
Male				
1	1999	2009	1.1 (0.7 to 1.5)^c^	3.2 (2.6 to 3.8)^c^
2	2009	2018	3.4 (2.8 to 4.0)^c^	
3	2018	2020	13.3 (7.3 to 19.5)^c^	
Female				
1	1999	2008	2.6 (2.1 to 3.2)^c^	4.9 (4.3 to 5.6)^c^
2	2008	2018	5.1 (4.5 to 5.7)^c^	
3	2018	2020	15.0 (8.1 to 22.4)^c^	
**Census region and sex**				
Northeast				
Male				
1	1999	2005	−2.2 (−3.1 to −1.4)^c^	2.2 (1.7 to 2.8)^c^
2	2005	2018	2.7 (2.4 to 3.0)^c^	
3	2018	2020	13.5 (7.8 to 19.5)^c^	
Female				
1	1999	2006	−0.6 (−2.6 to 1.4)	3.1 (2.3 to 3.9)^c^
2	2006	2020	5.0 (4.2 to 5.7)^c^	
Midwest				
Male				
1	1999	2007	−0.2 (−1.1 to 0.7)	3.5 (2.6 to 4.3)^c^
2	2007	2018	3.8 (3.2 to 4.5)^c^	
3	2018	2020	17.2 (8.1 to 27.1)^c^	
Female				
1	1999	2013	3.0 (2.2 to 3.8)^c^	5.1 (4.2 to 5.9)^c^
2	2013	2020	9.3 (7.0 to 11.7)^c^	
South				
Male				
1	1999	2011	−0.8 (−1.5 to −0.2)^c^	1.7 (1.2 to 2.2)^c^
2	2011	2020	5.1 (4.1 to 6.2)^c^	
Female				
1	1999	2010	0.4 (−0.7 to 1.4)	3.3 (2.5 to 4.0)^c^
2	2010	2020	6.6 (5.3 to 7.9)^c^	
West				
Male				
1	1999	2018	1.1 (0.9 to 1.3)^c^	2.0 (1.3 to 2.7)^c^
2	2018	2020	11.1 (3.2 to 19.6)^c^	
Female				
1	1999	2018	2.7 (2.4 to 3.0)^c^	3.6 (2.6 to 4.6)^c^
2	2018	2020	12.7 (1.7 to 24.9)^c^	

Abbreviations: AAPC, average annual percentage change; APC, annual percentage change.

^a^ APC measures the yearly rate of change within specific time segments, expressing the percentage change per year.

^b^ AAPC calculates the average rate of change over the entire study period, offering a perspective on the long-term trend and assessing the overall change in alcohol-related mortality.

^c^ *P* < .05; 95% CI does not include zero.

^d^ Hispanic individuals could be of any race; all other categories are non-Hispanic.

When stratified by race and ethnicity, recent trends in alcohol-related mortality were found to have increased in both male and female individuals. Non-Hispanic White individuals, non-Hispanic Black individuals, and American Indian or Alaska Native individuals showed higher recent trends among female individuals than male individuals. In contrast, Asian or Pacific Islander and Hispanic male individuals had higher trends than female individuals in the most recent time segment (Table 2).

Finally, when stratified by census region and sex, recent trends in alcohol-related mortality increased among both male and female individuals, but with differences in the rates of increase. In the Southern and Western regions, recent trends increased at a higher rate for male individuals than female individuals, while in the Northeastern and Midwestern regions, trends increased at a relatively higher rate for female individuals than male individuals (Table 2).

### Sensitivity Analysis

The analysis of alcohol-related mortality rates from 1999 to 2019 revealed distinct patterns. Initially, the rates remained relatively stable from 1999 to 2005 (APC, −0.2%; 95% CI, −1.4% to 0.3%), followed by a gradual increase at an annual rate of 1.7% (95% CI, 0.9%-2.8%) from 2005 to 2011 (Table 3). Subsequently, the rates accelerated significantly, with a more pronounced increase of 3.8% (95% CI, 3.5%-4.4%) per year from 2011 to 2019. On further examination by sex, both male and female individuals experienced increasing trends in alcohol-related mortality rates, but the rate of increase was higher among female individuals than among male individuals.

**Table 3.** Sensitivity Analysis: APCs and AAPCs in Alcohol-Related Mortality Rates by Sex, US, 1999-2019

Trend segment	Segment end points, y		APC (95% CI), %^a^	AAPC (95% CI), %^b^
	Lower	Upper		
Overall				
1	1999	2005	−0.2 (−1.4 to 0.3)	2.0 (1.9 to 2.1)^c^
2	2005	2011	1.7 (0.9 to 2.8)^c^	
3	2011	2019	3.8 (3.5 to 4.4)^c^	
**Sex**				
Male				
1	1999	2004	−1.1 (−2.4 to −0.4)^c^	1.5 (1.3 to 1.6)^c^
2	2004	2011	1.1 (0.5 to 1.8)^c^	
3	2011	2019	3.4 (3.0 to 3.9)^c^	
Female				
1	1999	2006	0.8 (−0.4 to 1.4)	3.1 (3.0 to 3.3)^c^
2	2006	2013	3.8 (1.5 to 4.4)^c^	
3	2013	2019	5.2 (4.5 to 7.0)^c^	
**Age and sex**				
15-24 y				
Male				
1	1999	2007	8.0 (4.5 to 14.0)^c^	0.7 (−0.5 to 1.9)
2	2007	2019	−4.0 (−6.4 to −2.2)^c^	
25-44 y				
Male				
1	1999	2005	−2.4 (−5.3 to −1.4)^c^	1.3 (1.0 to 1.5)^c^
2	2005	2012	0.5 (−0.7 to 3.0)	
3	2012	2019	5.3 (4.4 to 7.3)^c^	
Female				
1	1999	2005	−1.4 (−3.0 to 0.2)	2.9 (2.1 to 3.7)^c^
2	2005	2012	2.1 (0.4 to 3.7)^c^	
3	2012	2019	7.6 (6.3 to 9.0)^c^	
45-64 y				
Male				
1	1999	2006	0.3 (−0.7 to 0.8)	1.6 (1.5 to 1.7)^c^
2	2006	2019	2.4 (2.1 to 2.7)^c^	
Female				
1	1999	2007	2.6 (1.5 to 3.9)^c^	3.7 (3.4 to 3.9)^c^
2	2007	2015	5.0 (3.3 to 7.0)^c^	
3	2015	2019	3.0 (0.2 to 4.4)^c^	
≥65 y				
Male				
1	1999	2003	−2.8 (−4.8 to −0.7)^c^	1.3 (0.7 to 1.9)^c^
2	2003	2011	0.3 (−0.6 to 1.2)	
3	2011	2019	4.4 (3.6 to 5.2)^c^	
Female				
1	1999	2011	−0.5 (−1.4 to 0.1)	1.7 (1.3 to 2.1)^c^
2	2011	2019	5.2 (3.9 to 7.2)^c^	
**Race and ethnicity and sex** ^d^				
American Indian or Alaska Native				
Male				
1	1999	2005	−0.3 (−8.2 to 2.8)	2.5 (1.8 to 3.3)^c^
2	2005	2019	3.7 (2.6 to 8.5)^c^	
Female				
1	1999	2005	2.0 (−3.2 to 3.8)	3.9 (3.4 to 4.4)^c^
2	2005	2019	4.7 (4.2 to 7.0)^c^	
Asian or Pacific Islander				
Male				
1	1999	2019	1.7 (1.0 to 2.4)^c^	1.7 (1.0 to 2.4)^c^
Female				
1	1999	2003	−10.4 (−31.0 to 3.6)	2.0 (0.1 to 4.7)^c^
2	2003	2019	5.3 (2.4 to 16.1)^c^	
Black				
Male				
1	1999	2006	−6.4 (−8.2 to −5.5)^c^	−1.8 (−2.1 to −1.6)^c^
2	2006	2012	−1.5 (−4.0 to 0.4)	
3	2012	2019	2.6 (1.6 to 4.8)^c^	
Female				
1	1999	2007	−5.9 (−7.5 to −4.6)^c^	−0.6 (−1.0 to −0.2)^c^
2	2007	2019	3.1 (2.3 to 4.0)^c^	
Hispanic				
Male				
1	1999	2004	−3.5 (−7.9 to 0.7)	−0.4 (−0.9 to 0.1)
2	2004	2012	−0.5 (−4.2 to 4.1)	
3	2012	2019	1.9 (−2.7 to 6.3)	
Female				
1	1999	2004	−2.1 (−9.5 to 1.2)	1.2 (0.6 to 1.9)^c^
2	2004	2019	2.3 (1.6 to 5.2)^c^	
White				
Male				
1	1999	2005	0.6 (−1.3 to 1.6)	2.3 (2.1 to 2.5)^c^
2	2005	2011	2.0 (0.8 to 4.8)^c^	
3	2011	2019	3.8 (2.4 to 5.5)^c^	
Female				
1	1999	2009	2.8 (2.1 to 3.3)^c^	4.1 (3.9 to 4.3)^c^
2	2009	2019	5.5 (5.0 to 6.1)^c^	
**Census region and sex**				
Northeast				
Male				
1	1999	2005	−2.3 (−3.2 to −1.6)^c^	1.2 (1.1 to 1.4)^c^
2	2005	2019	2.8 (2.6 to 3.0)^c^	
Female				
1	1999	2006	−0.3 (−4.6 to 1.3)	2.8 (2.4 to 3.3)^c^
2	2006	2019	4.6 (3.8 to 5.8)^c^	
Midwest				
Male				
1	1999	2003	−1.6 (−4.6 to 0.1)	2.3 (2.1 to 2.6)^c^
2	2003	2011	1.8 (0.9 to 3.2)^c^	
3	2011	2019	4.8 (4.2 to 6.1)^c^	
Female				
1	1999	2011	2.7 (1.3 to 3.6)^c^	4.4 (3.9 to 4.9)^c^
2	2011	2019	7.1 (5.6 to 10.2)^c^	
South				
Male				
1	1999	2003	−2.1 (−4.2 to −0.8)^c^	1.1 (1.0 to 1.3)^c^
2	2003	2011	−0.2 (−0.7 to 3.2)	
3	2011	2019	4.2 (3.7 to 5.0)^c^	
Female				
1	1999	2003	−1.6 (−5.5 to 0.8)	2.6 (2.3 to 3.0)^c^
2	2003	2010	1.4 (0.1 to 7.4)^c^	
3	2010	2019	5.5 (3.0 to 8.2)^c^	
West				
Male				
1	1999	2006	0.3 (−1.7 to 1.0)	1.1 (0.9 to 1.3)^c^
2	2006	2019	1.5 (1.3 to 2.3)^c^	
Female				
1	1999	2006	1.5 (−1.4 to 2.5)	2.7 (2.4 to 2.9)^c^
2	2006	2019	3.3 (2.9 to 4.4)^c^	

Abbreviations: AAPC, average annual percentage change; APC, annual percentage change.

^a^ APC measures the yearly rate of change within specific time segments, expressing the percentage change per year.

^b^ AAPC calculates the average rate of change over the entire study period, offering a perspective on the long-term trend and assessing the overall change in alcohol-related mortality.

^c^ *P* < .05; 95% CI does not include zero.

^d^ Hispanic individuals could be of any race; all other categories are non-Hispanic.

## Discussion

Previous studies have provided valuable insights into the morbidity and mortality associated with alcohol in the US,^7,29,30^ helping us understand the burden and underlying factors involved. These studies highlight a higher prevalence of alcohol-related deaths among male individuals than among female individuals.^7,29,30^ Building on this existing knowledge, our analysis reveals a trend of increasing rates of alcohol-related deaths in both male and female individuals in recent years. However, female individuals have experienced a higher rate of increase compared with male individuals across different demographic categories, including race and ethnicity, age, cause, and region.

Our findings mirror the results of recent studies conducted in this area. For instance, a study examining alcohol-induced mortality trends from 2000 to 2016 found that the rates increased by 4.2% per year among men and 7.1% among women in the US.^5^ Another study by Angus et al^20^ explored the association of the COVID-19 pandemic with deaths related to alcohol, drugs, and suicide in the US from 2019 to 2021. They discovered that rates of alcohol-specific deaths were higher among women (29.1%) than men (26.7%) during this period. In addition, a study analyzing alcohol-related deaths in the US during the COVID-19 pandemic reported a greater increase in deaths among female individuals (27.3%) compared with male individuals (25.1%).^21^

The changing patterns of alcohol consumption among women are an important consideration in understanding these trends. Women are now drinking alcohol at higher amounts and frequencies than in the past, likely due to the normalization of alcohol use for female individuals in society.^8,9,10,11,12^ The change in the mortality rate trends perhaps parallels the changing patterns in general alcohol consumption as well as in disordered or harmful patterns of consumption (such as binge drinking) where the sex gap has also been closing globally.^9,10,31^ A study conducted among 2 nationally representative survey samples, comprising a total of 43 093 participants, found that women exhibited a greater increase than men in 12-month alcohol use, high-risk drinking, and *Diagnostic and Statistical Manual of Mental Disorders* (Fourth Edition) AUD.^9^ According to a meta-analysis of studies on birth cohort changes in male to female ratios in indicators of alcohol use as well as in alcohol-related harms, during the past century, there has been a steady decrease in male to female ratios for problematic alcohol use and alcohol-related harms, from approximately 3 to 1 among those born in the early 1900s to approximately 1 to 1 among those born in the late 1900s.^12^

The motivation for drinking is an important factor that may vary between male and female individuals and across age and race and ethnicity subgroups. Coping with stress is one of the main motivations for initiation of alcohol misuse for both male and female individuals.^32^ Stress also plays a major role in the development and maintenance of disordered drinking behaviors and, ultimately, addiction. In fact, development of AUDs is thought to be associated with distinctive neuroadaptations, including the upregulation in the brain stress system to counteract the effects of the chronic influx of dopamine release induced by persistent alcohol use.^33,34^ It is likely that the narrowing gap in sex differences for alcohol mortality rates, which also parallels the narrowing gap in the patterns of alcohol use and misuse,^12^ may be reflective of an increase in stress levels and stress-related disorders among women in recent decades and, particularly, in recent years.

Age, racial and ethnic, and regional differences were observed in sex-subtyped trends in alcohol-related mortality. Among adults aged 65 years or older, the rate of change in alcohol-related mortality was higher among female individuals than male individuals. This finding perhaps points to the larger burden of accumulating harms of chronic alcohol use among female individuals compared with male individuals rather than suggesting a higher amount of alcohol use by female individuals aged 65 years or older because the narrowing of the male-female gap is most prominent among young adults rather than adults aged 65 years or older.^12^

Recent mortality trends have increased at a higher rate among non-Hispanic White, non-Hispanic Black, and American Indian or Alaska Native women than men. Women in the Southern and Western census regions have recorded a higher increase than men in mortality rates in recent years. But, overall, the mortality rates in the Western census region are almost double that of any other census region for both male and female individuals. Despite the consistent pattern of the lowest rates of alcohol consumption,^35^ the Southern region showed comparable mortality rates with the Northeast and Midwest regions. These findings highlight the importance of addressing underlying factors as well as the interaction among factors associated with excessive alcohol consumption and alcohol-related harm, which may differ across age, regional, and race and ethnicity subgroups and also involve social, cultural, economic, and even religious factors that may be at play in shaping drinking habits of people at an individual level.

Given the rates of alcohol-related mortality, it is important to acknowledge the limited knowledge of how current pharmacologic treatments for AUD specifically affect women. As emphasized by McKee and McRae-Clark,^36^ the development of medications for AUD has often overlooked potential sex and gender differences. The US Food and Drug Administration–approved medications for AUD, such as disulfiram, naltrexone, and acamprosate, have been studied primarily in men, leaving a knowledge gap regarding their association with mortality outcomes in women.^36^ Although these medications have shown potential in improving health outcomes, their effectiveness in reducing alcohol-related mortality remains uncertain, particularly for women.^36^ For example, although naltrexone has demonstrated efficacy in reducing drinking and cravings, women may experience more adverse events, leading to higher rates of treatment discontinuation.^36^ Recognizing these gaps in understanding and considering sex and gender differences are crucial to developing interventions that target women’s alcohol use and have the potential to mitigate the rates of alcohol-related mortality among women.

Alcohol-related deaths in the US may have been associated with the COVID-19 pandemic, as well as with the observed sex differences.^20,21^ However, our sensitivity analysis demonstrated that our findings remained robust even when excluding data from the year 2020, with the latest trends increasing at a higher rate among women compared with men. Although our study sheds light on this trend, further research should be conducted to fully understand the underlying factors associated with the increased alcohol-related mortality among women. In addition, future studies should explore the potential association of the COVID-19 pandemic with alcohol-related deaths in more depth, considering various socioeconomic, psychological, and health care–related factors. Such investigations would help inform the development of targeted interventions and policies to address the growing public health issue of alcohol-related mortality, particularly among women.

### Limitations

This study has some limitations. It is primarily descriptive and does not explore the factors associated with alcohol-related mortality trends in both male and female individuals. Future research should incorporate predictive factors to provide a more comprehensive understanding of this public health issue. Another limitation is the restricted examination of age-specific trends, as well as the analysis of period and cohort effects. Due to data constraints, we were unable to delve deeply into these dimensions. A more detailed exploration of age-specific trends would have allowed for a better understanding of how alcohol-related mortality rates vary across different age groups. Moreover, investigating period and cohort effects could have provided valuable insights into the association of historical and generational factors with alcohol-related mortality rates. Future studies should address these limitations and provide a more nuanced understanding of how age, period, and cohort are associated with alcohol-related mortality rates. Finally, there were insufficient death counts for female individuals aged 15 to 24 years, which prevented us from calculating trends for this specific age range. Alternative data sources could be explored to bridge this gap and provide a more comprehensive analysis of alcohol-related mortality among female individuals in this age group.

## Conclusions

This cross-sectional study presents a comprehensive analysis of sex differences in alcohol-related mortality in the US from 1999 to 2020. Although male individuals continue to experience a higher burden of alcohol-related deaths, the findings suggest a trend of increasing rates of alcohol-related deaths among female individuals, indicating a narrowing sex gap. These trends may be associated with a combination of sociocultural, economic, biological, and behavioral factors, including the normalization of cultural practices surrounding alcohol consumption. Further research is necessary to identify the psychosocial and environmental factors associated with these trends and guide evidence-based interventions aimed at reducing alcohol-related mortality risks for all individuals, with a particular focus on developing targeted treatments to address alcohol use among female individuals.

## Notes

Supplement 1. **eTable.** *International Statistical Classification of Diseases and Related Health Problems, Tenth Revision* (*ICD-10*), Codes for Alcohol-Related Death Counts in the United States, 1999-2020

Supplement 2.
Data Sharing Statement
