# Supplementary material for: Trends in Alcohol-Related Deaths by Sex in the US, 1999-2020
Source: JAMA Netw Open. 2023 Jul 28;6(7):e2326346. doi: 10.1001/jamanetworkopen.2023.26346 (PMC10383009; doi:10.1001/jamanetworkopen.2023.26346)
Supplement: Supplement 1. — eTable. International Statistical Classification of Diseases and Related Health Problems, Tenth Revision (ICD-10), Codes for Alcohol-Related Death Counts in the United States, 1999-2020 [file jamanetwopen-e2326346-s001.pdf]

Supplementary Online Content

Karaye IM, Maleki N, Hassan N, Yunusa I. Trends in alcohol-related deaths by sex in the US, 1999-2020. *JAMA Netw Open.* 2023;6(7):e2326346.
doi:10.1001/jamanetworkopen.2023.26346

eTable. *International Statistical Classification of Diseases and Related Health Problems, Tenth Revision* (*ICD-10*), Codes for Alcohol-Related Death Counts in the United States, 1999-2020

This supplementary material has been provided by the authors to give readers additional information about their work.

© 2023 Karaye IM et al. *JAMA Network Open.*

eTable. *International Statistical Classification of Diseases and Related Health Problems, Tenth Revision* (*ICD-10*), Codes for Alcohol-Related Death Counts in the United States, 1999-2020

[a] ICD-10 Code	Description	Total number of alcohol-related deaths (N=605,948)
E24.4	Alcohol-induced pseudo-Cushing syndrome	N/A
F10	Mental and behavioral disorders due to use of alcohol	173, 933
G31.2	Degeneration of nervous system due to alcohol	2,989
G62.1	Alcoholic polyneuropathy	130
G72.1	Alcoholic myopathy	38
I42.6	Alcoholic cardiomyopathy	11,328
K29.2	Alcoholic gastritis	642
K70	Alcoholic liver disease	373, 302
K85.2	Alcohol-induced acute pancreatitis	5,203
K86.0	Alcohol-induced chronic pancreatitis	3,154
R78.0	Finding of alcohol in blood	40
X45	Accidental poisoning by and exposure to alcohol	33,190
X65	Intentional self-poisoning by and exposure to alcohol	815
Y15	Poisoning by and exposure to alcohol, undetermined intent	1,177

[a] ICD-10: International Classification of Diseases, 10th Revision Codes
[N/A] Mortality count unavailable due to data suppression constraints

© 2023 Karaye IM et al. *JAMA Network Open.*
