# Supplementary material for: Trends in Alcohol-Related Deaths by Sex in the US, 1999-2020
Source: JAMA Netw Open. 2023 Jul 28;6(7):e2326346. doi: 10.1001/jamanetworkopen.2023.26346 (PMC10383009; doi:10.1001/jamanetworkopen.2023.26346)
Supplement: Supplement 2. — Data Sharing Statement [file jamanetwopen-e2326346-s002.pdf]

Data Sharing Statement

Karaye. Trends in Alcohol-Related Deaths by Sex in the US, 1999-2020. *JAMA Netw Open*. Published July 28, 2023. doi:10.1001/jamanetworkopen.2023.26346

Data
Data available: Yes
Data types: Data (not involving human participants)
How to access data: https://wonder.cdc.gov/
When available: With publication

Supporting Documents
Document types: None

Additional Information
Who can access the data: The dataset is publicly available on the CDC WONDER webpage
Types of analyses: Any purpose
Mechanisms of data availability: The dataset is publicly available on the CDC WONDER webpage
Any additional restrictions: None
